# Extraosseous (extramedullary) plasmacytomas: a clinicopathologic and immunophenotypic study of 32 Chinese cases

**DOI:** 10.1186/1746-1596-6-123

**Published:** 2011-12-19

**Authors:** Zhuo Zuo, Yuan Tang, Cheng-Feng Bi, Wen-Yan Zhang, Sha Zhao, Xiao-Qing Wang, Qun-Pei Yang, Li-Qun Zou, Wei-Ping Liu

**Affiliations:** 1 Department of Pathology, West China Hospital of Sichuan University, Chengdu, China; 2 Department of Oncology, West China Hospital of Sichuan University, Chengdu, China

**Keywords:** Extraosseous plasmacytomas, extramedullary, clinicopathology, immunohistochemistry

## Abstract

**Background:**

Extraosseous plasmacytoma, so called extramedullary plasmacytoma (EMP) is relatively rare in China. The aim was investigate the clinicopathologic features of EMP and the role of Immunophenotype and genotype detection in diagnosis of EMP.

**Methods:**

Thirty-two cases of EMP were investigated retrospectively by histopathology, immunophenotype, genotype and survival analysis.

**Results:**

Clinically, the mean age of the patients was 53.4. Most of the patients received no treatment after the diagnosis was established, and the prognosis was relatively poor. Histologically, in 40% of the cases, the neoplastic cells were grade II or III. The neoplastic cells expressed one or more PC associated antigens. The immunophenotype of EMP and inflammation of sinonasal regions with numerous PC infiltrations were compared and showed some difference in expression of CD45, CD27, CD44v6 and Bcl-2 as well. Ig light chain restriction was detected in 87.5% of the cases.

**Conclusions:**

we described 32 Chinese cases of EMP, compare with that reported in the literature, some differences are presented, including higher percentage of grade II and III cases, clinically inconsistent treatment and management as well as poor outcome of the disease.

## Background

Extraosseous plasmacytomas, so called extramedullary plasmacytomas (EMP) is defined by the updated WHO classification (2008) as localized plasma cell neoplasms that arise in tissues other than bone [1]. EMP is a rare tumor and accounts for 3~5% of all plasma cell neoplasm. Approximately 80% of them occur in the upper aerodigestive tract. EMP has also been noted to arise in other anatomic sites, including the gastrointestinal tract, lymph node, bladder, CNS, breast, thyroid, testis, parotid and skin [1,2]. There were a few reports of the tumor in large numbers in literatures, and most of those studies mainly focused on clinical features and outcome analysis of the tumor as well [3,4]. Dores GM et al [5] reviewed and analyzed the incidence and survival for plasmacytoma of bone, EMP and multiple myeloma reported in the United States in period of 1992-2004. There were 1543 cases of plasmacytomas, and EMP occupied 30.7% (474/1543). In all 474 cases of EMP, most patients were white males with advanced age, only 41 of them were Asians. Furthermore, there is still no report of EMP coming from Mainland China in literature review now. In the period of January 1990 to February 2007, the total number of plasma cell neoplasms diagnosed in West China Hospital of Sichuan University (Chengdu, China) was 279, accounting for 13.97%(39/279) of all plasma cell neoplasms diagnosed in this period. In the current study, 32 cases of EMP were studied retrospectively for the clinicopathologic and immunophenotypic features of the tumor.

## Materials and methods

### Patient selection

Thirty nine patients with EMP were collected from the Pathology Department, West-China Hospital of Sichuan University in the period of January 1990 to February 2007. Seven of them were excluded from the study because of insufficient materials or problems of the clinical data. The remaining 32 cases were included in this study. Histological diagnosis of the tumors was based on WHO classification of the tumors of haematopoietic and lymphoid tissues (2008)[1] and HIV infection was not detected in all patients. Consistent methods used were as follows: (1) examination of histological features on slides of biopsies; (2) immunophenotype analysis on paraffin embedded tissue sections including B and T-cell differentiation antigens, universal plasma cell markers and some of related markers, as well as proliferation index; (3) Immunoglobulin (Ig) heavy chain gene (IGH) and light chain gene (IGK and IGL) rearrangement analysis by PCR. All cases selected fever the recommended diagnostic criteria for solitary EMP compiled by Soutar R, et al [6], they are as follows: (1) single extramedullary mass of clonal plasma cells; (2) histologically normal marrow aspirate and trephine; (3) normal results in skeletal survey, including radiology of long bone; (4) no anemia, hypercalcaemia or renal impairment due to plasma cell dyscrasia; (5) absent or low serum or urinary level of monoclonal immunoglobulin. The clinical features of these 32 patients, including age, gender, clinical course and follow-up data were compiled. In addition, 21 cases of solitary plasmacytoma of bone (SPB) and 12 cases of inflammatory lesions of aerodigestive tracts with numerous plasma cells were used as a control for immunohistochemistry and analysis.

### Histopathologic review

Routine H&E stained sections were used to assess the histopathologic features. The cases were graded according to the histological grading criteria described by Bartl et al [7] (1987). In addition, coagulation necrosis, amyloid deposition, blood cell lake as well as growth pattern of "starry sky" were also observed in morphologic review of the tumors. The mitotic index was estimated by counting the number of mitotic figures in 10 randomly selected high power fields(HPF) at 40 × 10 magnifications on a Leica 020-518.500 DM/LS microscope (Leica Microsystems Holding GmbH, Wetzlar, Germany).

### Immunohistochemistry analysis

The antibodies used include CD19 (LE-CD19, Dako, Denmark), CD20 (L26, Dako, Denmark), CD27 (137B4, Thermo, USA), CD38 (AT1, NeoMarker, USA), CD44v3 (VEF-327v3, NeoMarker, USA), CD44v6 (2F10, NeoMarker, USA), CD45 (PD7/26, Dako, Denmark), CD56 (123, C3, Zymed, CA), CD79α (JCB117, Dako, Denmark), CD117 (polyclonal, Dako, Denmark), CD138 (MI15, GeneTech, USA), PC (VS38C, Dako, Denmark), EMA (E29, Dako, Denmark), Bcl-2 (124, Dako, Denmark), CyclinD1 (SP4, Thermo, USA), kappa and lambda immunoglobulin (Ig) light chains (polyclonal, NeoMarker, USA) and Ki-67 (MIB-1, NeoMarker, USA). Four-micrometer thick sections were cut and deparaffinized in xylene and hydrated in a graded series of ethanol. The slides were then pretreated by pressure-cooking in citric acid buffer (10 mM, pH 7.4) for 3 minutes before staining for CD19, CD20, CD38, CD44v3, CD44v6, CD45, CD56, CD79α, PC, EMA, Bcl-2, in EDTA (1 mM, pH 9.0) for 8 minutes before staining for CD27, CD138, CyclinD1 and Ki-67 and in Trypsin (pH 7.8) for 10 minutes before staining for Igκ and Igλ light chains. Envision method was used for Immunohistochemical staining for CD19, CD20, CD45, CD79α, CD117, PC, EMA, Bcl-2, CyclinD1, Igκ, Igλ, and Elivision method was used for CD27, CD38, CD56, CD138, CD44v3, CD44v6, Ki-67. DAB was used as a substrate and the positive signal was dark brown in color.

### DNA extraction and polymerase chain reaction

Genomic DNA from paraffin-imbedded tissue samples was extracted by phenol-chloroform procedures. Successful DNA extraction was confirmed by amplification of 110 bp fragment of β-globin. Both universal IGH (FR3A/LJH/VLJH) primers and the BIOMED-2 PCR multiplex tubes were used for IGK and IGL rearrangement analysis. For universal IGH (FR3A/LJH/VLJH) detection, the major procedure was as follows: 1 μL of each primer at 25 μmol/L (Takara, Tokyo, Japan) were used in 25-μL reaction mixture. The PCR mixture (Takara, Tokyo, Japan) contained 2.5 μL of 10 × PCR buffer, 1.5 μL of MgCl2 25 mmol/L, 2 μl of dNTP 2.5 mmol/L of, 1.25 U of Taq DNA polymerase and 1 μg template DNA. Five microliters of PCR products was electrophoresed in 1.5% agarose gels (110 V, 30 min). Gels were stained with Goldview (Saibaisheng, China) and examined with ultraviolet light using the Image Acquisition and Analysis Software (Bio-Rad Laboratories, Herchles, CA). The BIOMED-2 PCR multiplex tubes (IgH, IGK and IGL) detection was according to the procedure of van Dongen et al [8].

### Survival and statistical analysis

Follow-up data were available in 15 of the 32 patients (46.9%). The overall survival time was plotted using the Kaplan-Meier (SPSS 13.0; SPSS, Chicago, IL). For comparison of the various parameters of immunohistochemistry of EMP, SPB and inflammatory lesions of nasal and sinonasal regions with numerous plasma cell infiltrations, χ2 test was used. A p value of 0.05 was considered significant.

## Results

### Clinical manifestations

**Figure 1:**
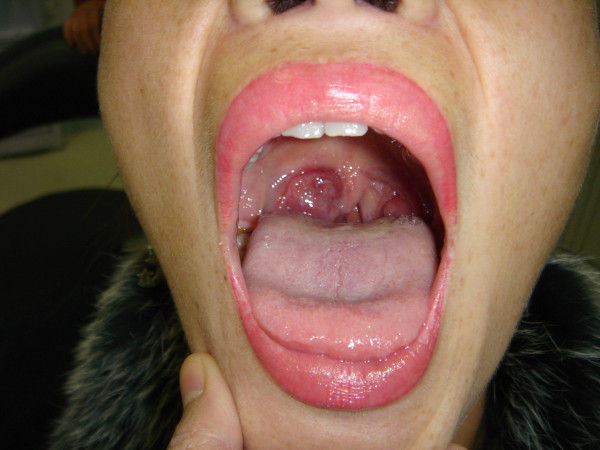
**A mass of EMP in oral cavity (case 31)**.

The clinical features of the patients are summarized in Table 1 and the manifestations in Table 2. The age of the patients ranged from 30 to 85 years and the mean age at diagnosis was 53.4 years. The male to female ratio was 2.2:1. The period to onset of the tumor was 14.1 months. The upper aerodigestive tract and tonsil were the most common sites of the disease, accounting for 72% of the cases, followed by skin (6 cases, 18.8%), gum, lung and testis (1case each). The patients displayed a solitary polyp like lesion in the upper aerodigestive tract (Figure 1) or nodule in subcutaneous or other solid organs. The size of the tumor ranged from 0.5 × 0.5 × 0.5 cm to 20.0 × 15.0 × 10.0 cm. The manifestations are closely related to the anatomic sites involved, e.g. in patients with nasal cavity or sinonasal lesions, the symptoms include nasal obstruction, rhinorrhoea and epistaxis. Laboratory examination did not show increased white cell count or plasmacytosis in all 32 cases. Serum and urine protein electrophoresis and immunofixation were performed in 21 and 8 patients, respectively. Low serum level of M-components was found in 2 of the 21 cases (9.5%), one of IgG type (21.4 g/L) and another of IgA type (4.36 g/L). whereas no M-protein of urine was found in all 8 patients examined. All patients received bone marrow aspiration or trephine and skeletal survey by standard X-ray and computed tomography (CT) or magnetic resonance imaging (MRI). No multiple osteolytic lesions were identified except for the solid mass in the involved regions. There was no evidence of bone marrow involvement at the time of diagnosis as well.

**Table 1:** Clinical features (n = 32)

Contents	Patients	
	No./Total number	Percentage
**Sex**		
Male	22	62.5
Female	10	37.5
**Age (years)**		
Range	30~85	
Mean	53.4	
Median	60	
**Sites**		
Upper areodigestive tract	21	65.6
Tonsil	2	6.3
skin	6	18.8
Gingiva	1	3.1
Lung	1	3.1
Testis	1	3.1
**Auxiliary examination**		
Normal marrow aspirate	32	100
Normal results on skeletal survey	32	100
low serum level of M-components	2/21	9.5
low urinary level of M-components	0/8	0
**Follow up (n = 15, month)**		
Dead	10/15	67 (6.6 m)
Alive with disease	3/15	20 (11.3 m)
Alive with recurrence	2/15	13 (24 m)

**Table 2:** Clinicopathologic features and follow-up data

**No**.	Age (years)	Sex	Sites	Size (cm)	Grading*	therapy	Follow-up (months)	
**1**	61	M	NC	1 × 1 × 1	II	S	Lost	
**2**	63	M	NC and PNS	8 × 5 × 1	II	S	Lost	
**3**	32	M	NC and PNS	2 × 2 × 2	II	S	Lost	
**4**	60	F	Lung	4.3 × 3.5 × 1.5	I	S	Lost	
**5**	47	M	NC	0.5 × 0.5 × 0.5	I	S	ANED	123
**6**	53	M	NC	1 × 1 × 1	I	S	Lost	
**7**	55	F	NC and PNS	5 × 3 ×1.5	I	S+RT+C	AWD	110
**8**	62	F	Chest wall	11 × 1 0	I	S	Lost	
**9**	63	M	Tonsil	3.5 × 3 × 2	I	S	AWD	97
**10**	54	M	NP	2 × 2 × 1	I	S	Lost	
**11**	46	M	Occiput	8 × 6 × 3	I	S	Lost	
**12**	49	F	NC	1.5 × 1 ×0.2	II	S	Lost	
**13**	48	M	NC	1.5 × 1 ×0.6	III	S	Died	?
**14**	58	M	NC and PNS	6 × 4 × 1	III	S	ANED	81
**15**	32	M	PNS	4.5 × 4 × 1.5	II	S	AWD	92
**16**	47	M	trunk (back)	20 × 1 5 × 1 0	I	S	AWD	75
**17**	34	M	NC and PNS	3 × 3 ×1	I	S	Lost	
**18**	50	F	NC	1 × 1 ×1	III	S	Lost	
**19**	66	F	NP	0.5 × 0.5 × 0.5	I	S+RT	ANED	65
**20**	77	M	Cervical	2 × 1.5 × 0.8	I	S	Lost	
**21**	35	M	NC and PNS	6 × 5 × 3	II	S	Lost	
**22**	47	M	NC and PNS	3 × 3 × 2	II	S	DOD	17
**23**	55	M	PNS	2 × 1.6 × 0.6	I	S	Died	?
**24**	63	M	PNS	3 × 1.6 × 0.8	I	S	DOD	51
**25**	67	F	gum	1.5 × 1.2 × 1	II	S	Lost	
**26**	65	M	cervicum	1 × 1 ×0.7	I	S	Lost	
**27**	85	F	forehead	2 × 1.4 × 1	I	S	DOD	11
**28**	56	M	PNS	6 × 5 × 1	III	S	DOD	228
**29**	30	M	testis	3.5 × 3 × 2	II	S	Lost	
**30**	56	M	NC	1 × 1 × 0.3	I	S	ANED	17
**31**	35	F	tonsil	1 × 1 × 1	I	S	AWD	6
**32**	59	F	PNS	5 × 4 × 3	II	S+RT	Lost	

M = male; F = female;

*Grading, according to Bartl *et al*. RT = Radiotherapy;

AWD = alive with disease; DOD = died of disease;

ANED = alive with no evidence of disease; NC = nasal cavity;

S = surgery; NP = nasopharynx;

**C = chemotherapy; PNS = paranasal sinus**.

R = relapse

### Histopathologic findings

**Figure 2:**
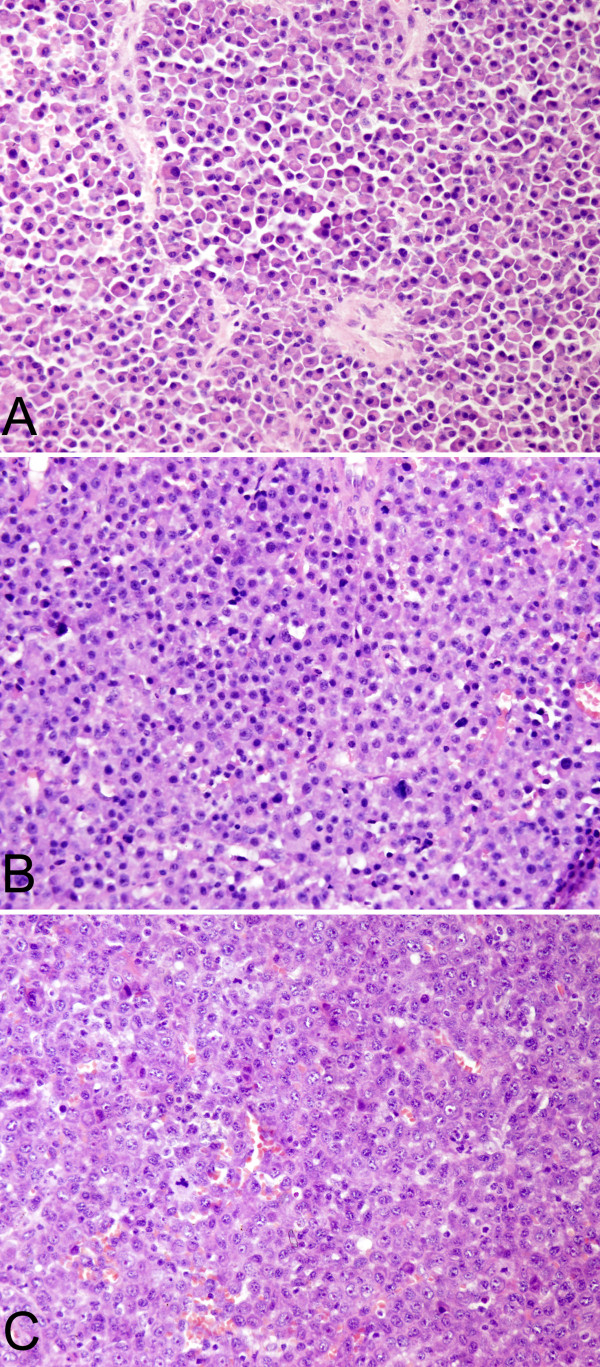
**Histopathologic features of EMP**. A. H&E stained section showed tumor cells of Grade I (case 27). B. Tumor cells of Grade II (case 22). C. Tumor cells of Grade III (case 28). D. Tumor cells infiltrated in subdermis (case 1).

**Figure 3:**
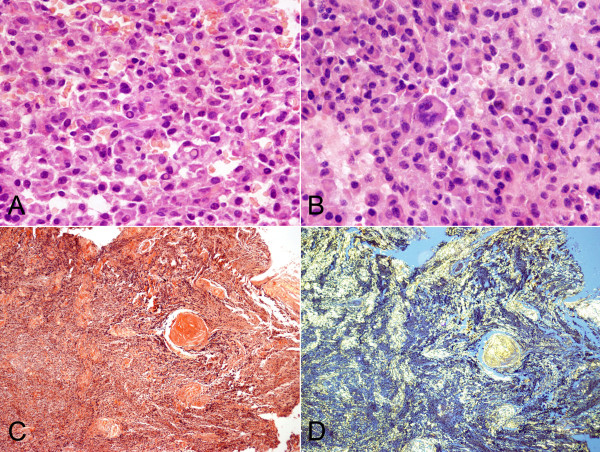
**Histopathologic features of EMP**. A. Dutcher's body (case 1). B. tumor giant cells (case 24). C. Amyloid deposition stained by congo red (case 9). D. Amyloid deposition stained by congo red showed a characteristic "apple-green" birefringence under polarized light (case 9).

The morphologic features of the cases are summarized in Table 3. According to histological grading criteria described by Bartl et al (1987), 18 of 32 cases (56.3%)were classified as well differentiated (grade I), 10 cases (31.3%) as moderately differentiated (grade II), and the remaining 4 cases (12.4%) as poorly differentiated (grade III). The neoplastic cells of grade I were similar to that of normal plasma cells and mitotic figure was rare. In contrast to the well differentiated EMP, the neoplastic cells of grade III often showed plasmablastic morphology exhibiting large nuclei with open chromatin and prominent nucleoli, mitotic figures were easily seen and tumor giant cells were also observed in 4 cases. Whereas the neoplastic cells of grade II were less homogeneous, and intermingled with normal looking plasma cells and anaplastic cells. Dutcher's bodies were seen in 4 cases (12.5%). Coagulation necrosis was visible in 11 of 32 cases (34.4%). Amyloid deposition with granulomatous reaction was found in 6 (18.8%). Blood cell lakes were presented 12 cases (37.5%). In addition, a "Starry Sky" pattern was observed in 3 cases (9.4%) (Figures 2 and 3).

**Table 3:** Morphologic features (n = 32)

Morphology	Number	Percentage
Tumor cell differentiation		
Well (Grade I)	18	56.3
Moderate (Grade II)	10	31.3
Poor (Grade III)	4	12.4
**Tumor giant cells**	10	31.3
**Dutcher's body**	4	12.5
**Necrosis**	11	34.4
**Amyloid deposition**	6	18.8
**Blood cell lake**	12	37.5
**"Starry Sky" pattern**	3	9.4

### Immunophenotype and genotype

**Figure 4:**
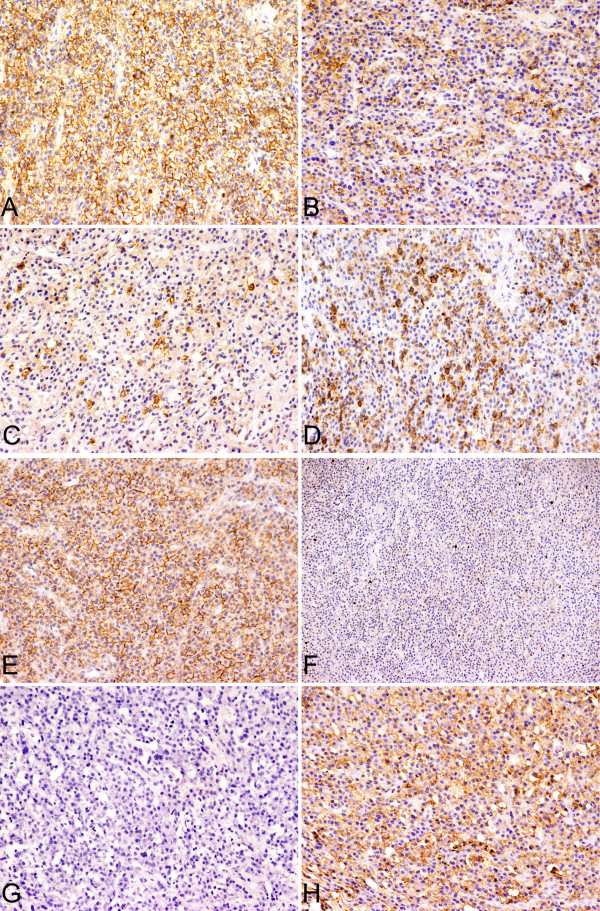
**Immunophenotype of EMP, Grade I (case 16)**. A. CD138. B. CD38. C. CD56. D. CD27. E. CD44v6. F. Ki67. G&H. Tumor cells showed the restricted lamppa chain expression.

**Figure 5:**
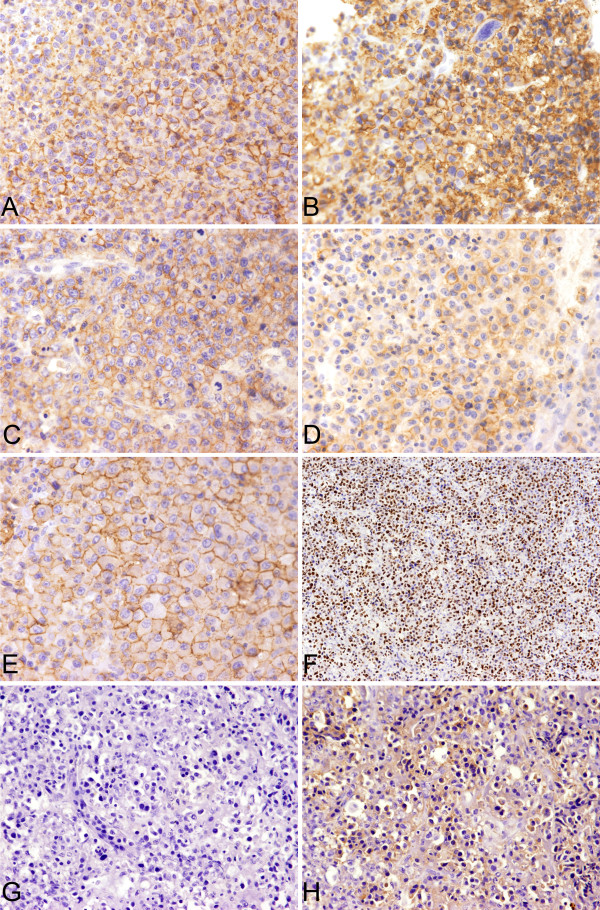
**Immunophenotype of EMP, Grade III**. A. CD138 (case 14). B. CD38 (case 18). C. CD56 (case 13). D. CD27 (case 28). E. CD44v6 (case 14). F. Ki67 (case 13). G&H. Tumor cells showed the restricted kappa chain expression (case 13).

The results of immunohistochemistry were summarized in Table 4. The neoplastic cells were positive for CD138 (100%), CD38 (90.6%) and vs38c (95.5%). 11 cases (34.4%) expressed CD45 antigen. Nearly 50% of the cases, the tumor cells expressed CD79a. In two cases some of tumor cells gave a positive reaction to CD20 or CD19, respectively. Bcl-2 expression was found in 31 cases (96.9%). The tumor cells expressed CD56 and Cyclin D1 protein in 6 cases each (18.8%). The expressions of CD117, CD27 and EMA were 28.1%, 43.8% and 31.3%, respectively. 56.3% and 11.5% of the cases expressed adhesion molecules of CD44v3 and CD44v6. Ig light chain restriction was observed in 28 cases (87.5%), including Igκ restriction of 11 cases (34.4%) and Igλ of 17 cases (53.1%). Ki-67 index was increased with the advanced histologic grading and ranged from 1 to 90% (Figures 4 and 5). The immunophenotype of EMP and inflammation of sinonasal regions with numerous PC infiltrations were compared and showed some difference in expression of CD45, CD27, CD44v6 and Bcl-2 and listed in Table 5. The difference in expression of CD56, CD27, CD44v6 and EMA between EMP and SPB were compared and showed in Table 6. Antigen receptor gene analysis was performed in 24 cases. Immunoglobulin heavy and light chain gene were clonally rearranged in sixteen of 24 cases (66.7%), including IGH of 9 cases (37.5%), IGK of 12 cases (50%) as well as IGL of 6 cases(25%).

**Table 4:** Immunophenotype (n = 32)

Antibody	Clonality	+/Total number	Percentage
**CD45**	PD7/26	11/32	34.4
**CD20**	L26	1/32	3.1
**CD19**	LE-CD19	1/32	3.1
**CD79a**	JCB117	15/32	46.9
**CD138**	MI15	32/32	100
**CD38**	AT1	29/32	90.6
**PC**	vs38c	23/24	95.8
**CD56**	123C3	6/32	18.8
*CD117	-	9/32	28.1
**EMA**	E29	10/32	31.3
**CD27**	137B4	14/32	43.8
**CD44v3**	VEF-327v3	3/26	11.5
**CD44v6**	2F10	18/32	56.3
**Bcl-2**	124	31/32	96.9
**CyclinD1**	SP4	6/32	18.8
*Igκ	-	11/32	34.4
*Igλ	-	17/32	53.1

* Polyclonal antibody

**Table 5:** The major phenotypic differences of EMP and chronic inflammation of nasal and sinonasal regions with numerous plasma cell (PC) infiltrations

Antibodies	EMP (n = 32)	**Chronic inf**. (n = 12)	*P value*
**CD45**	34.4%	100%	0.000
**CD27**	43.8%	100%	0.001
**CD44v6**	56.3%	0	0.011
**Bcl-2**	96.9%	0	0.000

**Table 6:** The major phenotypic differences of EMP and SPB

Antibodies	EMP (n = 32)	SPB (n = 21)	*P value*
**CD56**	18.7%	57.1%	0.004
**EMA**	31.3%	81.0%	0.000
**CD27**	43.8%	15.0%	0.032
**CD44v6**	56.3%	23.8%	0.020

### Treatment, follow-up, and survival analysis

Because most patients were treated in Outpatient Department of our hospital, the related data were unavailable or incomplete. Nevertheless, all patients received surgery for establishment of the diagnosis and/or initial treatment. Three patients (case 7, 19 and 32) received radiotherapy following surgery, and the total does was 45~65Gy. In addition, one patient (case 7) also received chemotherapy and the regimen was unknown.

Follow-up data were available for 13 patients (40.6%), and the other 2 died patients (6.25%), in whom the exact date and cause of death were all unknown at all. For the former 13 patients, the follow-up time ranged from 6 to 228 months, average of 74.8 months. 9 were grade I cases, and 4 were grade II and III. The overall survival time and the average survival time were 51 and 78.1 months, respectively. At the last follow-up, 4 patients died of the disease; 5 patients were alive with disease; and the remaining 4 patients were alive with complete remission (CR) as well. Furthermore, in 3 of the four patients living with CR, the size of the tumors was smaller (0.5 to 1 cm in diameter) and the histologic grading was lower (grading I) as well. No patient developed MM in process of the disease.

## Discussion

EMP is relatively rare, in our hospital, the total number of EMP diagnosed in period of 1990 and 2007 was 39, accounting for 13.97% (39/279) of all plasma cell neoplasms and 0.46% (39/8481) of all lymphoid neoplasms diagnosed during this period. Recently, Dores GM et al [5] reviewed plasma cell neoplasms reported in the literature in period of 1992-2004, and analyzed the incidence and survival for plasmacytoma of bone, EMP and multiple myeloma (MM) reported in the United States in this period. There were 1543 cases of plasmacytomas, and EMP occupied 30.7% (474/1543). In all 474 cases of EMP, most patients were white males with advanced age, only 41 of them were Asians. To date, the largest number of EMP (68 cases) was reported from Canada by Bachar et al [3] in 2008. The clinical features in current group of the cases are summarized as follows: (1) the patients were old, with a mean age of 60 years and male predominant; (2) in about 72% of the patients, the tumors were located in the midfacial region, and a high percentage of skin involvement was noticed; (3) most patients received no treatment, and the prognosis was relatively poor; (4) no development of MM was found. As a result, the general clinical manifestations of the patients are almost same as that reported in the literatures, including the age, gender, anatomic sites of the tumor and so on [2-4,6,9,10]. Talking about the treatment of the tumor, because the upper respiratory tract is most commonly involved, it is almost impossible to remove the tumor completely by operation, so that radiotherapy is often the first choices. No does-response relationship was observed now. The optimal radiation dose ranges from 40 to 50 Gy. If the tumor is greater than 5 cm in diameter, a higher does of radiation may be necessary for an effective treatment [6]. In the patients received radiotherapy, local recurrence rate of the tumor is usually less than 5%. Secondly, for the EMP of other anatomic sites, complete surgical removal should be considered if feasible. In addition, no recommendation for adjuvant radiotherapy can be made for patients who have undergone complete surgical excision with negative margins [6]. Bachar et al [3] reported 68 cases of solitary EMP of head and neck, the 5-year local recurrence-free rate (LRFR) was 81%, regional recurrence at 5 year was 5%, MM developed in 23% of the patients, and the 5-year survival rate was 76%. In current study, because most of our patients did not receive further treatment, after the diagnosis of EMP was established, their prognosis were not as good as that reported in the literature at all [5,6,11].

The histologic features of the cases in the current study are summarized as follows: (1) the neoplastic cells were moderately or poorly differentiated (grade II and III) in 40% of cases; (2) Variety degree of coagulative necrosis was observed in one third of the cases; (3) Amyloid deposition with granulomatous reaction and Dutcher's bodies were seen in 18.8% and 12.5% of the cases, respectively. Based on the report in literature, Most of EMP are well differentiated, the neoplastic cells are similar to normal plasma cells, especially the lesion of the upper aerodigestive tracts, there are often intermingled with variety of inflammatory cells, distinction between non-specific inflammation and plasma cell neoplasm is always problematic, Ig light chain restriction and Ig gene rearrangement analysis are useful in distinguishing non-neoplastic polyclonal proliferation from neoplastic clonal proliferation of plasma cells. In addition, because the morphologic changes of EMP are almost similar to those of extramedullary invasion of well differentiated MM, the diagnosis of EMP must be established on absolutely rule out the possibility of MM. The major clinical features of MM include hypercalcemia, renal insufficiency, anemia, multiple osteolytic lesions and so on. A constellation of clinical, laboratory, radiological and pathologic findings play an important role in diagnosis of MM as well. Sometimes, distinction from a marginal zone lymphoma with marked plasma cell differentiation is also a problem, particularly in skin and gastrointestinal tract, and may not be possible in some instance [1]. In contrast to the reports in literatures, higher percentage of grade II and III cases was noticed in current study, The differential diagnosis include the following neoplasms, such as large B-cell lymphoma (LBCL), poorly differentiated carcinoma, melanoma, as well as other types of sarcomas with features of epithelial differentiation. Immunohistochemistry will help a lot in distinction among those tumors. For LBCL, the neoplastic cells usually give a positive reaction to B-cell differentiation antigens, such as CD19, CD20, CD79a, PAX5 and so on, and usually negative for plasma cell markers of CD138 and CD38. Cytokeratins of different molecular weights are the useful markers for identification of various kinds of epitheliums. Sometimes, the neoplastic cells of poorly differentiated squamous cell carcinoma or adenocarcinoma, may also express CD138 or PC, it is necessary to choose a penal of antibodies in differentiation between carcinoma and EMP. The neoplastic cells of melanoma usually express HMB-45, MART-1 and S-100 protein, and it is easy to distinguish melanoma from EMP.

The immunophenotypic features of the cases in this study are summarized as follows: (1) the neoplastic cells of all cases expressed one or more plasma cell associated antigens; and CD138 and CD38 were better than PC in both sensitivity and specificity as well; (2) some difference of immunophenotypic expression in EMP, SPB and inflammation of sinonasal regions with numerous PC infiltrations were observed; (3) 87.5% of the cases displayed Ig light chain restriction, with Igλ type predominant; (4) Ki-67 index was elevated with the advanced histologic grading. In contrast to MM, there are limited reports giving a special observation for the immunophenotypic feature of plasmacytomas and their significance in literatures.

CD45, a key regulator of antigen mediated signaling and activation in lymphocytes, is present in early stages of PCs development. Kumar S et al [12] reported a total of 75 patients with untreated MM (29), relapsed MM (17), smoldering MM (12), and monoclonal gammopathy of undetermined significance (MGUS) (17), the proportion of PCs expressing CD45 was higher among those with early disease (MGUS or smoldering MM) compared to those with advanced disease (new or relapsed MM) (43 vs 22%; P¼0.005). Moreau P [13] reported that patients with CD45 negative MM receiving high-dose therapy have a shorter survival than those with CD45 positive MM. In this series of cases, 34.4% of EMP gave a positive reaction to CD45, whereas in 12 cases of inflammation of aerodigestive tracts, the infiltrated plasma cells were all positive for CD45.

Aberrant expression of CD56 is almost always observed in MM and more than 70% cases of MM express CD56 [14-16]. Few reports revealed that CD56 expressed in solitary plasmacytoma of bone (SPB) and in 10% EMP cases [4,17] Kermer M et al [4] compared the difference of Immunophenotype between EMP and MM. the results showed that EMP was infrequent expression of CD56, in contrast to both intra- and extra-medullary MM. In this series of cases, the percentage of CD56 expression was slightly higher than that reported in literature. We also observed CD56 expression in a group of inflammatory lesions of sinonasal regions with numerous plasma cell infiltrations; no PC gave a positive reaction to CD56 (the data did not show in this paper). The results suggested that CD56 may be used as a marker in differentiation between EMP and MM.

CD27 is a member of the still growing TNF receptor family [18,19] and is a marker of memory B cells, and its interaction with its ligand, CD70, is very important for differentiation into plasma cells [20-24]. Although CD27 is detected on normal plasma cells, its expression is significantly reduced with the progression of multiple myeloma (MM) [25-29]. MM patients in complete clinical remission displayed a higher percentage of CD27+ PC compared with patients at diagnosis, relapse or in partial remission [30]. No report has been found for CD27 expression in EMP yet. In current study, 43.8% of cases were positive for CD27.

CD44v6 is an isoform of the type 1 transmembrane glycoprotein CD44 generated by alternative splicing and has been implicated in cell adhesion and signaling, growth factor and cytokine presentation, as well as tumor dissemination [31-35]. Data on the incidence of CD44v6 in clonal plasma cell disorders are scarce. Liebisch et al [36] had observed CD44v6 expression in a group of plasma cell disorders, the results showed that CD44v6 was expressed in 13/32 in stage II/III MM correlated with chromosome 13q deletion, 1/6 in stage IA MM as well as 1/16 in MGUS. Therefore, the authors believed that CD44v6 is frequently expressed in advanced, high-risk MM. CD44v6 expression correlates with chromosomal band 13q14 deletions, a well known risk factor in MM. Eisterer et al [37] reported that CD44s was expressed strongly in all plasma cell populations included normal individuals, MGUS, MM, eMM and EMP. Although in 49 MMs, CD44v3 and CD44v6 expressed in 7 cases and 6 cases respectively, in 5 eMMs they were expressed in 3 cases and 4 cases respectively and in 2 EMPs they were expressed in all. In current series of cases, 56.3% of the cases expressed CD44v6, slightly higher than that reported in the literature. No difference of CD56, CD27 and CD44v6 expression was found in both low and high grade of EMP in current study at all.

The neoplastic cells of EMP and MM are positive for Bcl-2, whereas the reactive plasma cells usually do not express Bcl-2. Several authors also observed the relationship among CD45, Bcl-2 and Ki-67. No critical result was found now, in this study, nothing new was found as well.

## Competing interests

The authors declare that they have no competing interests.

## Authors' contributions

ZZ participated in the design, analyses and data interpretation and drafted the manuscript., YT, CFB, WYZ, SZ, XQW, QPY, LQZ collected pathologic and clinical information and helped to draft the manuscript. WPL conceived of the study and participated in its design and analyses and helped to draft the manuscript. All authors read and approved the final manuscript.
